# Detection of *Salmonella* Typhimurium with Gold Nanoparticles Using Quartz Crystal Microbalance Biosensor

**DOI:** 10.3390/s22228928

**Published:** 2022-11-18

**Authors:** Hyun Jung Min, Hansel A. Mina, Amanda J. Deering, J. Paul Robinson, Euiwon Bae

**Affiliations:** 1 Applied Optics Laboratory, School of Mechanical Engineering, Purdue University, West Lafayette, IN 47907, USA; 2 Department of Food Science, Purdue University, West Lafayette, IN 47907, USA; 3 Department of Basic Medical Sciences, Purdue University, West Lafayette, IN 47907, USA; 4 Weldon School of Biomedical Engineering, Purdue University, West Lafayette, IN 47907, USA

**Keywords:** quartz crystal microbalance, *Salmonella* Typhimurium, gold nanoparticles, frequency shifts

## Abstract

Demonstration of the *Salmonella* Typhimurium detection system was shown utilizing a quartz crystal microbalance (QCM) biosensor and signal enhancement by gold nanoparticles. In this study, a benchtop system of a QCM biosensor was utilized for the detection of *Salmonella* Typhimurium. It was designed with a peristaltic pump system to achieve immobilization of antibodies, detection of *Salmonella*, and the addition of gold nanoparticles to the sensor. As a series of biochemical solutions were introduced to the surface, the proposed system was able to track the changes in the resonant frequency which were proportional to the variations of mass on the sensor. For antibody immobilization, polyclonal antibodies were immobilized via self-assembled monolayers to detect *Salmonella* O-antigen. Subsequently, *Salmonella* Typhimurium was detected by antibodies and the average frequency before and after detecting *Salmonella* was compared. The highest frequency shifts were −26.91 Hz for $10^{9}\;\mathrm{CFU}/\mathrm{mL}$ while the smallest frequency shift was −3.65 Hz corresponding to $10^{3}\;\mathrm{CFU}/\mathrm{mL}$. For the specificity tests, non-*Salmonella* samples such as *E. coli*, *Listeria*, and *Staphylococcus* resulted in low cross-reactivity. For signal amplification, biotinylated antibodies reacted to *Salmonella* followed by streptavidin—100 nm AuNPs through biotin-avidin interaction. The frequency shifts of $10^{3}\;\mathrm{CFU}/\mathrm{mL}$ showed −28.04 Hz, and consequently improved the limit of detection.

## 1. Introduction

A quartz crystal microbalance (QCM) is a mass-based biosensor that measures a shift in a resonant frequency of a quartz crystal caused by an accumulated mass on the surface of the quartz crystal. The quartz crystal is one of the piezoelectric materials that operate based on a piezoelectric effect. The QCM technique uses an inverse piezoelectric effect that generates mechanical deformation when the voltage is applied. Due to these advantages of the quartz crystal, QCMs have been utilized to monitor film deposition [1,2], explore molecular interactions [3,4], and detect hazardous gases [5,6]. In food safety, Muramatsu et al. first developed a piezoelectric immunosensor with an AT-cut piezoelectric crystal for the detection of microbial pathogens in 1986 [7]. Afterwards, QCMs have been used to detect foodborne pathogens [8,9,10], viruses [11,12,13], and mycotoxins [14,15,16].

The operation of the QCM can be broken down into three parts: the bioreceptor, the design of signal acquisition, and methods for signal amplification. First, it is important to select an appropriate bioreceptor as well as an immobilization method suitable for the specific target. Monoclonal IgA was proven to be a more suitable bioreceptor for aflatoxin than IgG because it has twice the molecular weight of monoclonal IgG, allowing a higher detection range in QCM systems [14]. Monoclonal antibodies generally provide high specificity, but antibodies are more expensive than other counterparts while polyclonal antibodies have the possibility of cross-reactivity due to multiple epitopes but provide more inexpensive options [17,18]. Lectins were used as a recognition of targets where a lectin-based QCM with a flow-injection assay format was developed to detect *C. jejuni* [19]. Several lectins such as Concanavalin A (ConA), Ulex europeus (UEA), Maackia amurensis (MAL), Lens culinaris (LCA), and Triticum vulgaris (WGA) were explored as bioreceptors to find a strong affinity to the target. A ConA-based QCM was able to show the largest frequency shifts and detect $10^{3}$ cells of the *C. jejuni* strain in about 30 min. Aptamers could be an alternative for a bioreceptor because aptamers have several advantages such as low cost and long-term stability. Aptamers against *Salmonella* Typhimurium were investigated as bioreceptors detecting $10^{3}\;\mathrm{CFU}/\mathrm{mL}\;$ of *Salmonella* Typhimurium [10]. In the QCM system, it is necessary to compare the mechanism of bacterial attachment to the surface because this is closely related to the resonant frequency shift. Olsen et al. observed that a tight attachment of bacteria results in a negative frequency shift that was predicted by the Sauerbrey equation. The somatic O-antibody provides rigid binding compared to other attachment sites while flagellar H7 antibodies provide more degrees of freedom and form flexible attachments [20]. Therefore, somatic antibodies were recommended on acoustic wave sensors to build the tight attachment of bacteria and minimize a weak binding. 

The latest trends in biosensors have been towards high sensitivity, quantitative analysis, and portability thanks to the application of recent technologies such as the internet of things (IoT), and nanomaterials. In food safety, the sensitivity of the QCMs is an important factor because low concentrations of pathogens need to be detected. In particular, nanomaterials have been widely used to enhance the signal in the QCM biosensors and they play the role of a label as well as improving the signal in the immunoassays [21]. The most popular metal nanoparticle is AuNP due to its good biocompatibility and unique optical properties [22]. For instance, streptavidin-conjugated nanoparticles in a protein A-based QCM sensor helped to lower the limit of detection of *E. coli* O157:H7 by five orders of magnitude [23]. In addition, micro/nanobeads with different materials and sizes (i.e., magnetic, silica, and polymer) were investigated to amplify the frequency signal caused by *E. coli* O157:H7 using the QCM sensor [24]. Magnetic beads with a diameter of 350 nm were found to provide the largest frequency change, detecting the *E. coli* ranging from $10^{0}\;\mathrm{CFU}/\mathrm{mL}\;$ to $10^{6}\;\mathrm{CFU}/\mathrm{mL}$. Viruses with particles were detected as well due to the use of signal amplification methods. Influenza A and B viruses were detected with the application of AuNPs in the QCM system, showing a detection limit of $10^{3}\;\mathrm{pfu}/\mathrm{mL}$ [25]. Moreover, the QCM, combined with the polymerase chain reaction (PCR), detected the vaccinia virus DNA by amplifying the virus DNA [26]. The captured DNA was immobilized on the 10 MHz quartz crystal using biotin and NeutrAvidin binding. It showed a low number of amplification rounds for detection of the virus and the analysis time was around 15 min. Most recently, Lalit M. Pandey suggested designs of the engineered surface of QCM-based techniques to detect the severe acute respiratory syndrome coronavirus 2 (SARS-CoV-2) [27].

In this study, a benchtop QCM system combined with a fluidic system was developed to detect *Salmonella* Typhimurium by comparing the frequency shifts and increasing the signal by conjugating AuNPs. The frequency drop was monitored in real time and the comparison before and after *Salmonella* and AuNPs was performed. Polyclonal antibodies specific to O-antigen were investigated as a bioreceptor. The self-assembled monolayer (SAM) method was described for the immobilization of antibodies on the quartz crystal for the application of an antigen–antibody reaction. Tests for non-*Salmonella* samples were conducted for cross-reactivity. Finally, AuNPs were investigated to achieve a lower limit of detection by amassing the weight. A biotin–avidin complex was used to attach AuNPs to the cells of the *Salmonella*. The biotinylated antibody reacted to the *Salmonella* as secondary antibodies following streptavidin AuNPs conjugates. 

## 2. Materials and Methods

### 2.1. Theoretical Background

In 1959, Sauerbrey first demonstrated that additional mass on the surface resulted in a proportional decrease in the resonant frequency in air or a vacuum [28]. The Sauerbrey equation is valid if it is a rigid and uniform film and can be expressed as

(1) $$-C\frac{\Delta f}{n}=\Delta m$$

where Δ*f* is a resonant frequency shift (Hz) of the resonator, *C* is the mass sensitivity constant, *n* is the number of overtones and Δ*m* is a change in mass. The higher resonant frequency of the quartz crystal has a higher sensitivity and therefore more frequency shifts could be shown with the higher resonant frequency. Kanazawa and Gordon reported an equation when a quartz crystal was in contact with a Newtonian liquid in the 1980s [29]. This relationship is expressed as

(2) $$\Delta f_{k}=-f_{0}^{\frac{3}{2}}\sqrt{\frac{\rho \mu }{\pi \rho_{q}\mu_{q}}}$$

where $f_{0}$ is a resonant frequency of the unloaded crystal, *ρ* is the density of a liquid, *μ* is the viscosity of the liquid, and *ρ_q_* and *μ_q_* refer to the quartz’s density and viscosity. 

**Figure 1:**
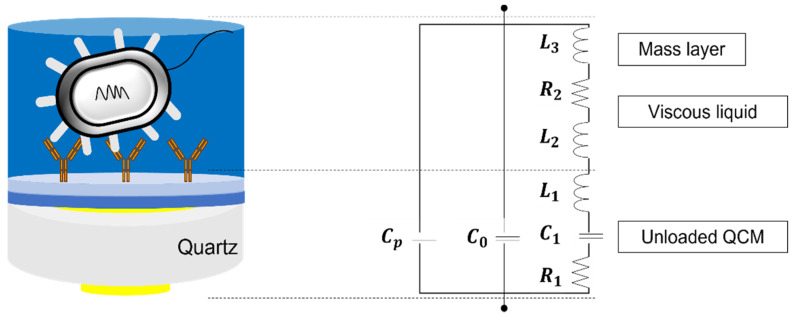
A quartz crystal in liquid media can be modeled as an electric circuit which is the Butterworth—Van Dyke equivalent circuit model. An unloaded QCM itself can be expressed by $R_{1}$, $L_{1}$, $C_{1},\;C_{0}$ and $C_{p}$. When it is in liquid media, the electric circuit is extended by adding additional $L_{2}$ and $R_{2}$ terms for the viscous liquid and $L_{3}$ for the thin rigid mass.

Figure 1 illustrates an electrical model of a quartz crystal which is an unloaded BVD model and a loaded BVD model [30]. In the simplest form, the unloaded BVD model consists of a parallel capacitance ($C_{0}$), inductance ($L_{1}$), and capacitance ($C_{1}$). $C_{0}$ is a static capacitance of the electrodes and $C_{p}$ is a parasitic capacitance of the test fixture. $L_{2}$ and $R_{2}$ can be calculated by the properties of the liquid and the parameter $L_{3}$ can be calculated by the properties of the mass [31].

### 2.2. A Benchtop QCM System

**Figure 2:**
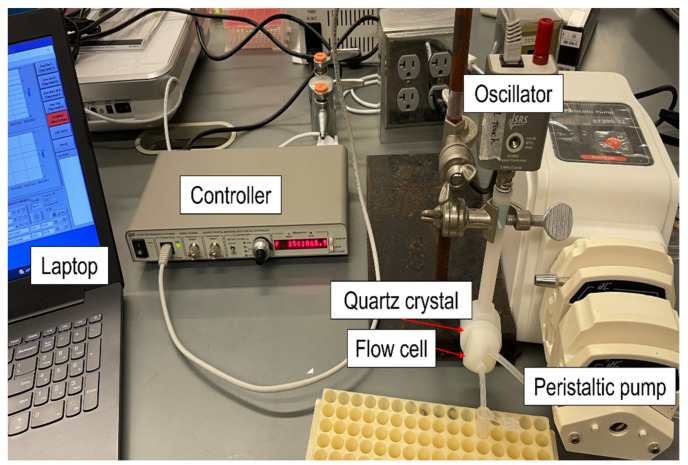
A photo of a QCM benchtop setup for bacteria detection. A controller is connected to an oscillator and a laptop to receive the frequency data. A quartz crystal is installed in a flow cell, and a peristaltic pump allows the solution to be introduced to the front side of the quartz crystal.

A benchtop QCM system consists of a 5 MHz quartz crystal (O100RX1, Stanford Research Systems, Sunnyvale, CA, USA), a QCM digital controller (QCM200, Stanford Research Systems, Sunnyvale, CA, USA), and an oscillator (QCM25, Stanford Research Systems, Sunnyvale, CA, USA) (Figure 2). The QCM digital controller was connected to the laptop (Lenovo, Beijing, China) while a resonant frequency with a resolution of 0.01 Hz was displayed on the front panel and saved on the laptop simultaneously. On the other side of the controller, the oscillator was connected to the back side of the 5 MHz quartz crystal through two spring-loaded contacts. The quartz crystal was positioned vertically inside a flow cell (O100FC, Stanford Research Systems, Sunnyvale, CA, USA) to transfer the solution into the front side of the quartz crystal and to minimize the occurrence of air bubbles. The volume of the flow cell was about 150 μL and the solution was injected in the direction of the middle of the quartz crystal. A peristaltic pump (BT100-2J + 2YZ15, Robotdigg, HK, China) was used and the adjustable speed range was 0.1–100 rpm. Each solution was transferred through the pump in a 1 mL tube.

### 2.3. Biochemical Reagents

11-Mercaptoundecanoic acid (MUA), EDC (1-ethyl-3-(3-dimethylaminopropyl) carbodiimide hydrochloride), and NHS (N-hydroxysuccinimide) were purchased from Millipore Sigma, and Thermo Fisher Scientific. One mM of 11-MUA was dissolved in ethanol while 5 mM of EDC and 5 mM of EDC were prepared in water. 0.01 M phosphate-buffered saline including 0.138 M NaCl and 0.0027 M KCl (PBS, pH 7.4) was prepared to wash unnecessary things and measure the frequency to provide the same condition. One percent of bovine serum albumin (BSA)-PBS (pH 7.4) was prepared to block the unbound surface after immobilizing the antibodies. Goat anti-*Salmonella* CSA-1 antibody and biotin-labeled anti-*Salmonella* CSA-1 antibody were purchased from the same company, LGC seracare (Milford, MA, USA). Streptavidin-100 nm AuNPs conjugates were purchased from cytodiagnostics and 100 μL of AuNPs stock solution was diluted in PBS to use the final volume of 500 μL while the concentration was 0.15 mg/mL. Milli-Q water was used for all reagents throughout.

### 2.4. Procedure for Antibody Immobilization, Salmonella Detection and Application of AuNPs

**Figure 3:**
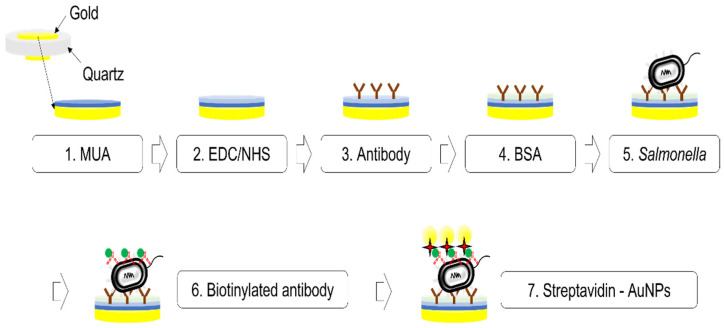
A procedure for antibody immobilization, *Salmonella* detection, and signal amplification. Each step was processed continuously and the PBS washing step was included between each step to remove unbounded residue.

Figure 3 illustrates the overall procedure of *Salmonella* detection which was divided into three steps: antibody immobilization, *Salmonella* detection, and signal amplification using gold nanoparticles. For the antibody immobilization, the SAM method was formed by thiols. First, 500 μL of 11-MUA was treated on the gold surface to form thiol groups, producing a strong affinity. After the flow cell was filled with 11-MUA, followed by PBS washing, the pump was stopped to provide enough reaction time (approximately one hour). Second, 500 μL of EDC/NHS was also introduced to form the NHS ester and stopped approximately for an hour, followed by PBS washing. Third, following EDC/NHS, antibodies specific to *Salmonella* of 200 μg⁄mL were immobilized for 1 h to connect the active NHS ester to the primary amines of that antibody. In the fourth stage, the non-specific binding surface was blocked with 500 μL of 1% BSA-PBS for 30 min. In the fifth stage, 1 mL of pure *Salmonella* introduced by the method in 2.5 was prepared for different concentrations, injected into the surface, and detected by the antibodies. The concentration ranged from $10^{3}\;\mathrm{CFU}/\mathrm{mL}\;$ to $10^{9}\;\mathrm{CFU}/\mathrm{mL}\;$ and for each set, a brand new QCM sensor was utilized. Triplicates of testing were conducted for each concentration while one control data represents pure PBS without *Salmonella*. For the signal amplification, a biotin-streptavidin interaction was utilized by 500 μL of biotinylated antibodies of 100 μg⁄mL and 500 μL of streptavidin-AuNPs of 30 μg⁄mL. Between the steps, the treated surface was washed with PBS to remove unbound materials, and the flow cell was filled up with PBS to measure the frequencies. All the experiments were performed at room temperature.

### 2.5. Acquisition of Frequency Data

All frequencies were measured in real time. After flowing each solution, the peristaltic pump was paused for around 50 min to provide enough reaction time. The frequencies for comparisons were obtained at a stop mode in the PBS of the pump before and after injection of BSA, *Salmonella* Typhimurium, biotin Ab, and streptavidin- AuNPs. Between each step with different material injection, PBS was used to remove the residue and wash any loose binding material, allowing less disturbance at oscillation. At a stop mode, we waited for 10 min to acquire the stable measurement of frequency, and the frequencies of the last three minutes were averaged before the next solution for comparison.

### 2.6. Bacterial Strains and Culture Preparation

*Salmonella enterica* ser. Typhimurium ATCC 14028 for standard tests and *E. coli* O157:H7 B6-914, *Staphylococcus aureus* ATCC 12600 and *Listeria monocytogenes* 10403S for the specificity tests were prepared according to the Bacteriological Analytical Manual [32,33,34]. First, cultures of *Salmonella* Typhimurium., *E. coli* O157:H7, *Staphylococcus,* and *L. monocytogenes* were grown in a Luria–Bertani broth (LB broth) and in a brain heart infusion broth (BHI broth), respectively, for 24 h at 32 °C in a shaking incubator at 150 rpm until they reached a final concentration of about $10^{9}\;\mathrm{CFU}/\mathrm{mL}$ after achieving an optical density (OD_600_) corresponding to 0.36, 0.67, 0.59, and 0.58, respectively. Second, centrifugation was used at 4226× *g* for 5 min for the samples and the bacterial pellets were then resuspended and washed in 30 mL of 0.1 M phosphate buffer, pH 7.0 (PB) three times. Aliquots with concentrations from $10^{3}\;\mathrm{CFU}/\mathrm{mL}$ to $10^{9}\;\mathrm{CFU}/\mathrm{mL}$ were obtained by serial dilution using the PBS solution. Finally, 100 µL of culture was spread on selective media such as XLT4, MacConkey sorbitol, Mannitol salt agar, and Oxford Medium, for *Salmonella* Typhimurium, *E. coli* O157:H7, *Staphylococcus*, and *Listeria*, respectively. After 36 h of incubation at 32 °C, the number of bacterial colonies was counted. For QCM experiments, 1 mL bacteria were heat-treated at 100 °C in a dry bath for 10 min, then stored at 4 °C until use.

### 2.7. Scanning Electron Microscope (SEM)

*Salmonella* and AuNPs were captured by the antibody immobilized on the QCM surface. Imaging was performed using 5 kv and spot size 3 with the Nova Nano SEM (FEI Company, Hillsboro, OR, USA). Sensors were fixed overnight in 2.5% glutaraldehyde in 0.1 M cacodylate buffer, post-fixation 2% osmium tetroxide, followed by ethanolic dehydration from 50% through 100% and finally dried with HMDS (hexamythldisalizane).

## 3. Results

### 3.1. SEM Images

**Figure 4:**
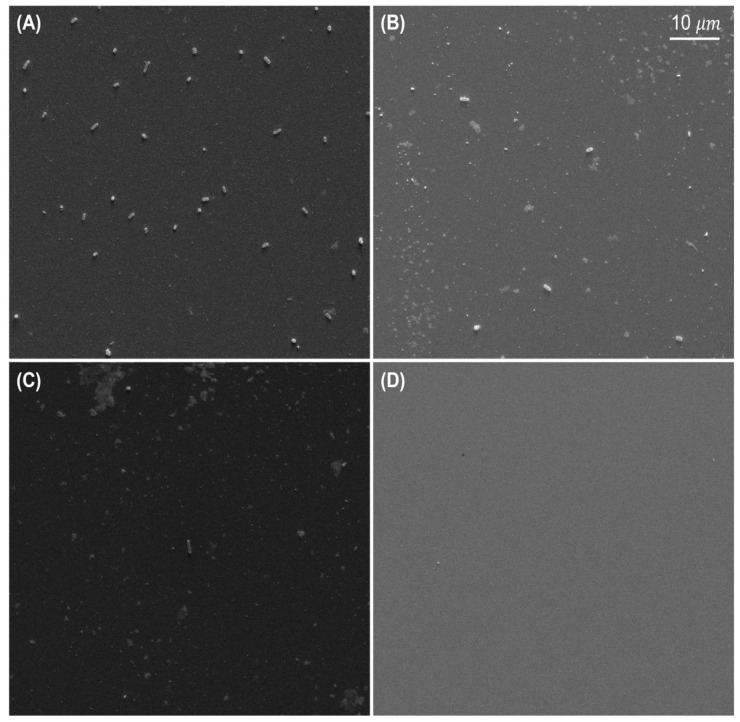
SEM images on the surface of QCM. Concentrations of *Salmonella* were (**A**) $10^{9}\;$ CFU/mL, (**B**) $10^{7}\;$ CFU/mL, (**C**) $10^{5}\;$ CFU/mL, and (**D**) a clear quartz crystal surface, respectively. They were present on the surface with different populations.

Figure 4A–D show the *Salmonella* cells corresponding to the concentration of $2\times 10^{9}\;\mathrm{CFU}/\mathrm{mL},$ $2\times 10^{7}\;\mathrm{CFU}/\mathrm{mL}$, $1.5\times 10^{5}\;\mathrm{CFU}/\mathrm{mL}$, and control (PBS), respectively, on the quartz crystal surface which has gone through the process shown in Figure 3.

**Figure 5:**
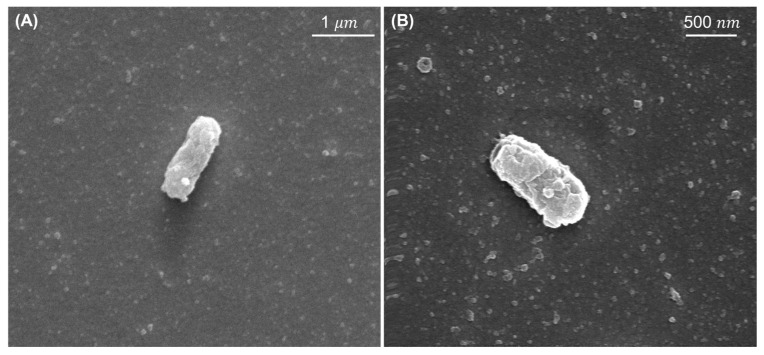
*Salmonella* with 100 nm-AuNPs were captured with a scale bar of 1 μm (**A**) and 500 nm (**B**). AuNPs were attached via a biotin-streptavidin reaction.

The number of *Salmonella* counted was 26, 7 and 1 for $2\times 10^{9}\;\mathrm{CFU}/\mathrm{mL},$ $2\times 10^{7}\;\mathrm{CFU}/\mathrm{mL}$, and $1.5\times 10^{5}\;\mathrm{CFU}/\mathrm{mL}.$ Within the given viewing area ($60\times 50\;{\mu m}^{2}$), the total number of *Salmonella* cells on the gold surface could be counted based on the number of *Salmonella* captured by SEM images in the given area. For example, Figure 4C showed a single cell corresponding to $1.5\times 10^{5}\;\mathrm{CFU}/\mathrm{mL}$ and the estimated concentration was $1.33\times 10^{4}\;\mathrm{CFU}/\mathrm{mL}$ obtained by extrapolating from the SEM image. There is an order of magnitude difference between the actual concentrations and the total number because *Salmonella* were spreading over the sensor, and they are not homogeneously positioned on each $60\times 50\;{\mu m}^{2}$ area segment. Figure 5 illustrated closer images of *Salmonella* to investigate 100 nm AuNPs. One or two 100 nm AuNPs were attached to the single cell.

### 3.2. Real-Time Frequency Monitoring for Bacteria Tests

**Figure 6:**
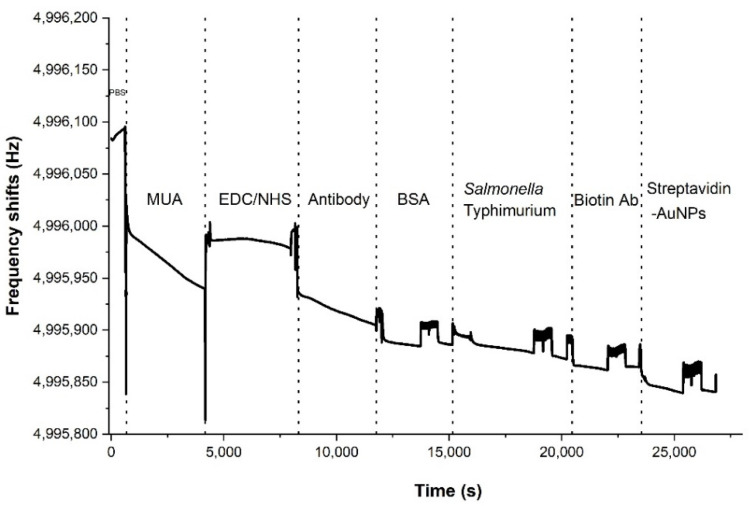
Total resonant frequency responses for a single measurement result from the whole procedure for *Salmonella* detection. Each material was indicated when the solution was replaced.

Figure 6 shows the real-time resonant frequency response of the whole process including antibody immobilization, *Salmonella* detection, and signal amplification, which took about 7 h. However, the resonant frequency was measured every second in real time, which means it was possible to track the frequency drop caused by the presence of bacteria at a flow mode and a stop mode. To investigate the accurate frequency shifts without weak binding or other unbound materials, the frequency was measured at a stop mode after washing with PBS. Measuring the *Salmonella* took around 1.5 h, while the attachment of AuNPs took 1.75 h. After the sensor was exposed to each material, the shift in the resonant frequency of the sensor showed a downward trend representing an incremental increase of mass loading on the sensor.

**Figure 7:**
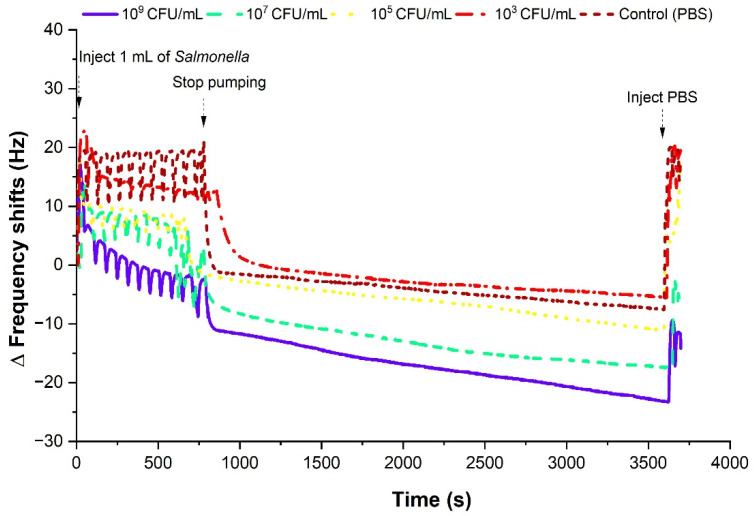
Frequency responses immediately after *Salmonella* Typhimurium injection. Large gaps can be observed between the high and low concentration due to the change in mass. Each graph represents a single measurement.

Figure 7 examined the resonant frequency response from the starting point of injection of the *Salmonella,* at a stop mode, and the injection of PBS. As the concentration of *Salmonella* Typhimurium decreased, the frequency shift also decreased with respect to the baseline frequency shifts. While injecting 1 mL of *Salmonella* with 0.3 rpm at the first 660 s period, the frequency gradually dropped with different gradients depending on the concentrations. After it was stopped and we waited for 1 h, larger gaps at a stop mode were shown than the flow mode between 0 s and 660 s.

**Figure 8:**
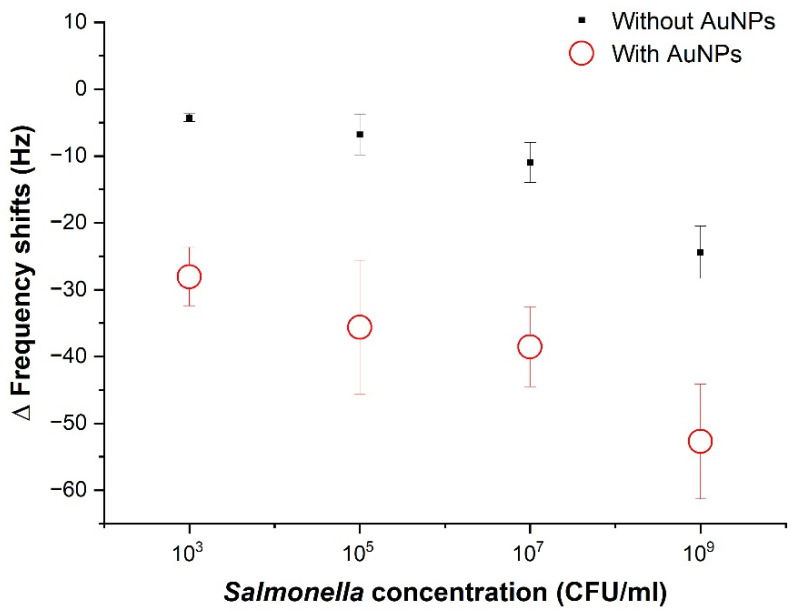
Comparisons of the average frequency shifts with and without AuNP attachment to the target bacterial cells (*n* = 3). The black points indicate the average frequency shifts before and after *Salmonella* while the red points indicate the average frequency shifts before and after *Salmonella* and AuNPs.

Figure 8 shows comparisons of the average frequency before and after injecting *Salmonella* from $10^{3}\;\mathrm{CFU}/\mathrm{mL}$ to $10^{9}\;\mathrm{CFU}/\mathrm{mL}$ and after AuNPs based on the method illustrated in Figure 3. Positive correlations were observed between the *Salmonella* concentration with and without AuNPs (Table 1). The highest frequency shift was −24.4 Hz after the *Salmonella*, corresponding to $10^{9}\;\mathrm{CFU}/\mathrm{mL}$, while the frequency shift of −4.27 Hz was recorded for $10^{3}\;\mathrm{CFU}/\mathrm{mL}$. The limit of detection (LOD) of *Salmonella* Typhimurium was estimated at somewhere between $10^{3}-10^{5}\;\mathrm{CFU}/\mathrm{mL}$ without AuNPs while the LOD after AuNPs is $10^{3}\;\mathrm{CFU}/\mathrm{mL}$. This is because S/N of larger than 2 is a conventionally accepted criterion. Signal to noise (S/N) is 1.5 and 2.4 at $\;10^{3}$ and $10^{5}\;\mathrm{CFU}/\mathrm{mL}$ without AuNPs while S/N is 2.5 and 3.2 at $\;10^{3}$ and $10^{5}\;\mathrm{CFU}/\mathrm{mL}$, respectively, with AuNPs. This is a similar trend to that in *E. coli* O157:H7 [35]. Mao et al., observed −30 Hz for $10^{7}$ and $\;10^{8}$ CFU/mL and −15 Hz for $10^{3}\;\mathrm{CFU}/\mathrm{mL}$, showing the detection limit $2.67\times 10^{2}\;\mathrm{CFU}/\mathrm{mL}$ using 8 MHz quartz crystal [35].

To increase the mass and thus further lower the LOD, AuNPs were accumulated via the strong interaction between biotinylated antibodies and streptavidin AuNPs. The use of AuNPs resulted in the additional mass, increasing the frequency shifts, thus improving the LOD. The highest frequency shift of $10^{9}\;\mathrm{CFU}/\mathrm{mL}\;$ with AuNPs was 52.72 Hz, while the frequency shift of $10^{3}\;\mathrm{CFU}/\mathrm{mL}$ was −28.05 Hz. As a result, an increase in sensitivity was more dominant in the lower concentration samples which resulted in a 123.74%, 268.14%, 471.94%, and 573.19% increase in frequency shifts corresponding to $10^{9}\;\mathrm{CFU}/\mathrm{mL},10^{7}\;\mathrm{CFU}/\mathrm{mL},10^{5}\;\mathrm{CFU}/\mathrm{mL}$ and $10^{3}\;\mathrm{CFU}/\mathrm{mL}$, respectively. The LOD after AuNPs was $10^{3}\;\mathrm{CFU}/\mathrm{mL}$. In the previous report, Jiang et al. found around −50 Hz for *E. coli* O157:H7 of $10^{6}\;\mathrm{CFU}/\mathrm{mL}$ with 350 nm magnetic nanobeads using 7.995 MHz [24]. The concentration from $10^{3}\;\mathrm{CFU}/\mathrm{mL}$ to $10^{6}\;\mathrm{CFU}/\mathrm{mL}$ showed the linear relationship with the frequency shifts. Liu et al. also observed a notable change around −15 Hz from *E. coli* O157:H7 of $10^{7}$ CFU/mL [23].

### 3.3. Specificity Test

**Figure 9:**
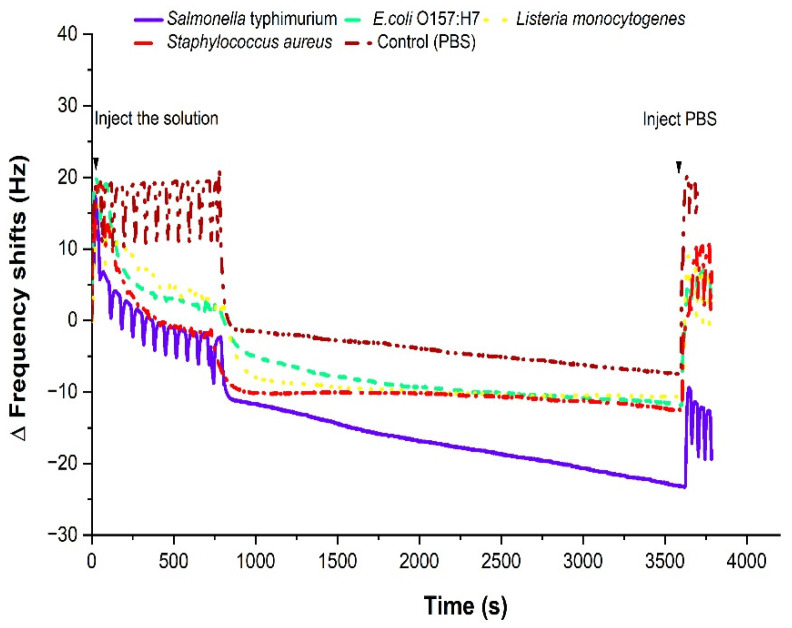
Frequency responses immediately after non-*Salmonella* such as *E. coli* O157:H7, *Listeria monocytogenes*, and *Staphylococcus aureus*. The concentration was $10^{8}–10^{9\;}\mathrm{CFU}/\mathrm{mL}.$.

Specificity tests are an important feature of the biosensor that reliability and selectivity. An Antigen−antibody reaction is used to capture a specific antigen so that the ability of the sensor can be determined by the characteristics of antibodies. To investigate the specificity of the sensor, pure non-*Salmonella* samples were challenged. With the same antibody immobilization method, *Salmonella* was replaced by four different genera of bacteria: *E. coli* O157:H7, *Listeria monocytogenes*, and *Staphylococcus aureus.* Figure 9 indicates the frequency responses over time for control, *Salmonella*, and non-*Salmonella* samples. The frequency drops were shown at a flow mode and at a stop mode after injecting *Salmonella* or non-*Salmonella* with respect to the baseline (control sample). The vertical axis displays the frequency drop for *E. coli* O157:H7, *Listeria monocytogenes*, *Staphylococcus aureus*, and *Salmonella*, which is evidence of the antibody−antigen reaction.

While we were expecting negligible frequency shifts from non-*Salmonella* samples, non-zero frequency shifts were observed for all negative samples due to the cross-reactivity of the antibody.

Furthermore, a frequency shift was monitored for the addition of AuNPs for the non-*Salmonella* samples as well. Table 2 contains the comparison of frequency shifts before and after non-*Salmonella* samples only (without AuNPs), and after biotinylated antibodies and streptavidin- AuNPs (With AuNPs). The frequency shift of $10^{5}\;\mathrm{CFU}/\mathrm{mL}$ of *Listeria monocytogenes* with AuNPs was −11.55 Hz, which was very similar to −10.9 Hz from the control sample. Furthermore, positive shifts in frequency were observed for *E. coli* O157:H7 and *Staphylococcus aureus* with AuNPs. This can be explained as the removal of existing bacteria that were captured by the antibody which had a weak bonding for non-*Salmonella* samples. With continuous flowing of AuNPs, these particles can separate the cells from the antibody, thus generating a positive frequency shift.

## 4. Discussion

Since QCM is a mass-based sensor, a mass of a single bacteria cell can be a starting point for discussion. Assuming that a single cell is 665 fg [36] and the total number of cells is distributed rigidly and uniformly on the active crystal area, the Sauerbrey equation that is commonly used to interpret the behavior of quartz crystal and the Butterworth−Van Dyke (BVD) model could be applied to investigate the relationship between frequency shifts and the total mass (Figure 1). When the total number of cells is $10^{5}$, the series frequency shifts showed −28 Hz for the Butterworth–Van dyke (BVD) model by changing the inductance value with fixed values R and C values (Figure S1). Meanwhile, −9.4 Hz was obtained from the Sauerbrey equation. However, bacteria cells in a liquid environment cannot be interpreted merely by resonant frequency. The recently proposed Kelvin–Voigt model and Maxwell model are a revised equation of the Sauerbrey model to interpret bacterial adhesion and biofilm maturation [37]. However, it is difficult to apply in a biological system since Young’s moduli obtained with the bacterial properties cannot be obtained [38]. Therefore, mathematical models of bacterial cells need to be developed.

Quartz crystal microbalance biosensors provide a rapid and in situ analysis over the conventional method for bacteria detection [39,40]. In recent years, many bacteria detection methods have been focused on the development of biosensing elements such as aptamer, DNA, etc. to increase the sensitivity and specificity. An aptamer-based digital PCR chip showed 90 CFU/reaction for *S. Typhimurium* cells while the method still has the limitation of capture efficiencies and multiplexing. Loop-mediated isothermal amplification (LAMP) also showed the LOD of $2.1\times 10^{2}\;\mathrm{CFU}/\mathrm{mL}$ of the *Salmonella* genus [41]; however, it largely relies on the selection of the target sequence and specificity of the primer. A fluorescent platform of DNA-stabilized silver nanoclusters showed 82 CFU/mL of LOD for *S.* Typhimurium while the equipment is still bulky and requires complex steps [42]. Even though the proposed method could not reach as high an LOD as other current technologies [42,43,44], the QCM has the potential of a handheld device that is able to detect the bacteria in real time as a bacteria detection method. Additionally, the sensitivity can be improved by the quartz crystal itself with a higher resonant frequency and the other biosensing elements applied.

Ideally, antibodies on the sensor should recognize only *Salmonella* spp. which means no frequency shifts should be detected when other bacteria are challenged. However, the proposed QCM result (without AuNPs) in Table 2 showed non-zero frequency shifts higher than the control, as shown in Table 1. This is attributed to the characteristics of the polyclonal antibodies. Based on the manufacturer’s performance specification of the antibodies, their antibodies can have a low cross-reactivity to *E. coli*: O157, and *Staphylococcus aureus*. Interestingly, the frequency shift in the lower concentration ($10^{5}\;\mathrm{CFU}/\mathrm{mL}$) for *Listeria* was −7.62 Hz, which was similar to −7.96 Hz from the higher concentration ($10^{9}\;\mathrm{CFU}/\mathrm{mL})$ sample. This indicates that cross-reactivity of the *Listeria* to our antibody was independent of the concentration or that the *Listeria* could have relatively stronger binding to the antibody so that the subsequent washing steps did not remove the weakly bound cells.

Table 1 proves that the sensitivity of the QCM with 100 nm-AuNPs was improved by increasing the frequency shifts. However, the frequency shifts caused by AuNPs were not proportional to the cell concentrations, producing little difference between different concentrations. One possible reason for this phenomenon is that AuNPs electrostatically repel each other. Even though a higher concentration of cells should capture more AuNPs, due to a relatively large number of AuNPs in the flow mode, they repelled each other which resulted in a similar level of frequency drops from $10^{3}\;\mathrm{CFU}/\mathrm{mL}$ to $10^{9}\;\mathrm{CFU}/\mathrm{mL}$. Ly et al. commented that when the size of AuNPs is larger, the repulsive force is stronger [45]. The size from 21 to 30 nm yielded the amplified frequency shifts proportional to the size while the size from 30 to 63 nm decreased the signal. Liu et al. reported that the frequency shifts caused by 145 nm-AuNPs were not proportional to the concentrations of *E. coli* cells [23]. They mentioned that this was because biotinylated antibodies may adsorb onto the Au electrode surface and protein A that was used for antibody immobilization. Jiang et al. also investigated different sizes and materials of micro/nanobeads that might generate different strengths [24]. Silica beads with diameters of 970 nm and 520 nm were found to have a positive frequency shift while 350 nm magnetic beads showed the largest negative shifts. The results did not indicate any relationship between the size and frequency shifts.

In this study, the frequency shifts after secondary biotinylated antibodies injection were proportional to the increased *Salmonella* concentrations. This means biotinylated antibodies react to *Salmonella* as the concentrations increase. The positive frequency shifts shown in specificity tests (Table 2) can be also explained by this characteristic. The repelling force of AuNPs caused the weak bonding of non-*Salmonella* to be washed off during a flow mode or even immobilized the chemical compounds [46]. Therefore, it could be deduced that sizes or materials may affect the frequency shifts. Furthermore, a coupled resonance model may be used to interpret this phenomenon. According to the coupled resonance model, particle adhesion leads to rather positive frequency shifts depending on the resonance frequency of the attached particles. However, the theory contradicts the Sauerbrey theory. When a crystal with resonance (*ω*) is higher than the adhering spheres with resonance frequency $\left(\omega_{s}\right)$, a positive frequency shift is calculated [47,48]. In contrast, a negative shift is obtained when the $\omega_{s}$ is higher than the ω that is expected. In the case of bacteria detection, this phenomenon can occur due to fimbriae and proteins on the bacterial surface [49]. In the same way, bacteria cells together with AuNPs may be interpreted by the coupled resonance model. To summarize, this study found that the effects of AuNPs are not proportional to the cell concentrations and further investigation of sizes or materials of nanoparticles as mass enhancers will be needed.

## 5. Conclusions

To detect *Salmonella* Typhimurium, a benchtop system of QCM was designed with the peristaltic pump system before developing a portable QCM device. Antibodies were immobilized to capture the different concentrations of *Salmonella* Typhimurium. while the real-time frequency response was monitored. For signal amplification, a biotin-streptavidin interaction allowed AuNPs to be added to the *Salmonella* as a mass, resulting in the increase in the frequency shifts and lowering in the limit of detection which is $10^{3}\;\mathrm{CFU}/\mathrm{mL}$. Therefore, the benchtop QCM system could detect the lower concentration of *Salmonella* with AuNPs, revealing a potential tool for bacteria detection.

## Figures and Tables

**Table 1:** Comparisons between *Salmonella* concentrations from plate counting method and frequency shifts data. The unit is Hz. SD represents standard deviation calculated based on Bessel’s correction.

*Salmonella* Concentration (CFU/mL)	Without AuNPs		Biotin Ab	With AuNPs	Total Δf (B)	Percentage Change $\frac{\left(B-A\right)}{A}\times 100\;\left(\%\right)$
	Δf (A)	SD				
$10^{9}$	−24.40	3.9	−15.13	−28.32	−52.72	123.74
$10^{7}$	−10.96	3	−7.38	−27.60	−38.57	268.14
$10^{5}$	−6.78	3.05	−4.44	−28.85	−35.63	471.94
$10^{3}$	−4.27	0.58	−4.13	−23.78	−28.05	573.19
Control (PBS)	−2.82		−2.89	−8.08	−10.9	

**Table 2:** Results of specificity tests. After non-*Salmonella*, negative frequency shifts were shown. After AuNPs, positive frequency shifts were observed. The results indicated that they might be washed off after the washing steps.

Non-Salmonella	Concentration (CFU/mL)	Average Frequency Shifts (Δf) after Non-*Salmonella* (Hz)		Total Δf
		Without AuNPs	With AuNPs	
*E. coli* O157:H7	$10^{9}$	−9.12	9.65	0.53
*Listeria monocytogenes*	$10^{8}$	−7.96	28.33	20.37
*Listeria monocytogenes*	$10^{5}$	−7.62	−3.93	−11.55
*Staphylococcus aureus*	$10^{9}$	−7.2	4.92	−2.28

## Supplementary Materials

The following supporting information can be downloaded at: https://www.mdpi.com/article/10.3390/s22228928/s1, Figure S1. Estimated frequency shifts of different bacteria concentrations based on the Butterworth van dyke model. The $10^{3}$ is almost same when the cell number is 0. As increasing the cell numbers, the frequency shifts are higher due to the change in the inductance in the electrical model.
