# Supplementary material for: Detection of Salmonella Typhimurium with Gold Nanoparticles Using Quartz Crystal Microbalance Biosensor
Source: Sensors (Basel). 2022 Nov 18;22(22):8928. doi: 10.3390/s22228928 (PMC9697148; doi:10.3390/s22228928)
Supplement: Supplementary file 1 [file sensors-22-08928-s001.zip › sensors-1995823-supplementary.pdf]

Detection of *Salmonella* Typhimurium with Gold Nanoparticles Using Quartz Crystal Microbalance Biosensor

Hyun Jung Min [1], Hansel A. Mina [2], Amanda J. Deering [2], J. Paul Robinson [3,4] and Euiwon Bae [1,*]

[1] Applied Optics Laboratory, School of Mechanical Engineering, Purdue University, West Lafayette, IN 47907, USA
[2] Department of Food Science, Purdue University, West Lafayette, IN 47907, USA
[3] Department of Basic Medical Sciences, Purdue University, West Lafayette, IN 47907, USA
[4] Weldon School of Biomedical Engineering, Purdue University, West Lafayette, IN 47907, USA
* Correspondence: ebae@purdue.edu

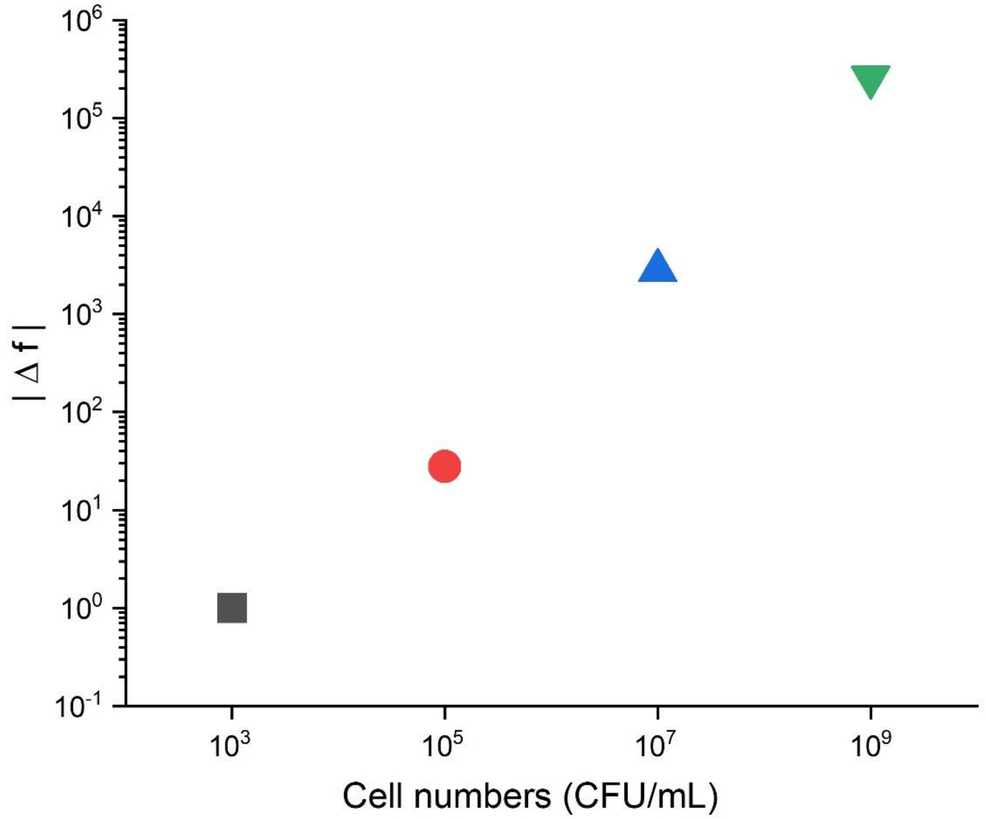

Figure S1. Estimated frequency shifts of different bacteria concentrations based on the Butterworth van dyke model. The 10^3 is almost same when the cell number is 0. As increasing the cell numbers, the frequency shifts are higher due to the change in the inductance in the electrical model.
